# Highly sensitive single-molecule detection of macromolecule ion beams

**DOI:** 10.1126/sciadv.adj2801

**Published:** 2023-12-01

**Authors:** Marcel Strauß, Armin Shayeghi, Martin F. X. Mauser, Philipp Geyer, Tim Kostersitz, Julia Salapa, Oleksandr Dobrovolskiy, Steven Daly, Jan Commandeur, Yong Hua, Valentin Köhler, Marcel Mayor, Jad Benserhir, Claudio Bruschini, Edoardo Charbon, Mario Castaneda, Monique Gevers, Ronan Gourgues, Nima Kalhor, Andreas Fognini, Markus Arndt

**Affiliations:** ^1^ Faculty of Physics and Vienna Doctoral School of Physics (VDSP) and Vienna Center for Quantum Science and Technology (VCQ), University of Vienna, Boltzmanngasse 5, A-1090 Vienna, Austria.; ^2^ Institute for Quantum Optics and Quantum Information (IQOQI) Vienna, Austrian Academy of Sciences, Boltzmanngasse 3, A-1090 Vienna, Austria.; ^3^ MSVision, Televisieweg 40, 1322 AM Almere, The Netherlands.; ^4^ Department of Chemistry, University of Basel, St. Johannsring 19, CH-4056 Basel, Switzerland.; ^5^ Advanced Quantum Architecture Laboratory, EPFL, Rue de la Maladière 71b, CH-2002 Neuchâtel, Switzerland.; ^6^ Single Quantum, Rotterdamseweg 394, 2629 HH, Delft, The Netherlands.

## Abstract

The analysis of proteins in the gas phase benefits from detectors that exhibit high efficiency and precise spatial resolution. Although modern secondary electron multipliers already address numerous analytical requirements, additional methods are desired for macromolecules at energies lower than currently used in post-acceleration detection. Previous studies have proven the sensitivity of superconducting detectors to high-energy particles in time-of-flight mass spectrometry. Here, we demonstrate that superconducting nanowire detectors are exceptionally well suited for quadrupole mass spectrometry and exhibit an outstanding quantum yield at low-impact energies. At energies as low as 100 eV, the sensitivity of these detectors surpasses conventional ion detectors by three orders of magnitude, and they offer the possibility to discriminate molecules by their impact energy and charge. We demonstrate three developments with these compact and sensitive devices, the recording of 2D ion beam profiles, photochemistry experiments in the gas phase, and advanced cryogenic electronics to pave the way toward highly integrated detectors.

## Teaser

Superconducting nanowires are highly efficient detectors for beams of massive proteins even at low impact energy.

## INTRODUCTION

Mass spectrometry is a versatile tool in the life sciences, chemistry, and physics, allowing the detection, identification, and analysis of objects ranging in size from atoms to large biopolymers. Some of the most widespread instruments, such as quadrupole mass spectrometers (QMSs) (*1*) and time-of-flight mass spectrometers (TOF-MSs), use detectors that rely on secondary electron multiplication (SEM). While SEM detectors have a quantum yield of up to η≃ 90% for electrons (*2*), the efficiency for macromolecules depends strongly on their velocity, mass, and structure (*3*, *4*) and can drop to η ≃ 10^−5^ for proteins with kinetic energies of only a few tens of electron volts. It only becomes large for impact velocities of *v* > 20 km/s, i.e., the impact energy of 40 keV for masses beyond 20 kDa (*5*). Since more than 80% of all known proteins are found in this mass regime (*6*), and singly charged proteins have attracted increasing interest (*7*), efficient detectors are clearly desirable.

The detection challenge is alleviated when working with highly charged ions since their kinetic energy is proportional to their charge *q* and the accelerating electric potential *U*_acc_. On the other hand, a low charge state is often favorable as it reduces the complexity of the mass spectrum and prevents spectral overcrowding in regions of low *m/z* values. In that case, detectors are required that can efficiently detect ions at impact velocities below 200 to 500 m/s. The capability to analyze biomolecules at long interaction times can also open new analytic opportunities in combination with optical spectroscopy and deflectometry, for instance, to determine dipole moments or polarizabilities, eventually even as a function of conformation (*8*). Here, we explore superconducting nanowires as quantum detectors for biomolecules at energies about 100 times smaller than commonly required for SEM detection, providing the same or even better spatial resolution, with the potential to improve on that by more than an order of magnitude.

Superconducting detectors are intriguing as they have a small energy gap in the few milli–electron volt regime that allows them to be highly sensitive to low-energy quanta. This gap can be bridged by the impact energy of a particle or by the absorption of a photon. A variety of cryogenic sensors have found interdisciplinary application in numerous research fields in the form of bolometers (*9*), transition edge sensors (*10*, *11*), kinetic inductance detectors (*12*), superconducting tunneling junctions (*13*, *14*), or superconducting nanowire detectors (SNWDs), also known as superconducting single-particle detectors (SSPDs) (*15*, *16*). All these sensors have important use cases in photonics, and a few of them have also been successfully applied in TOF-MS (*14*), including transition edge sensors (*10*), superconducting tunnel junctions, and SSPDs.

In comparison to superconducting tunnel junctions, SSPDs combine good spatial and temporal resolution with a working temperature above 3 K, where substantially higher cooling power is available at moderate cost (*17*). This is important for scaling current prototypes to multi-pixel devices, which are advantageous for applications that require large detector areas or high spatial resolution. SSPDs are typically etched into a superconducting film in a meander structure on top of a silicon-based substrate, as described in Materials and Methods. During operation, the nanowire is driven by a bias current *I*_b_ close to its critical current, typically in the 10- to 100-μA range. If a particle affects the detector, then the energy released to the superconductor locally breaks up Cooper pairs, causing a quantum phase transition from the superconducting to the normal conducting state. As a result, a normal conducting hot spot is generated. When the current around the resistive area exceeds the local critical current density, the strip will become normal conducting along its full width. The continuous driving of a current into the now resistive strip leads to a fast voltage peak across a shunt resistor that triggers the signal. Thermal relaxation to the substrate resets the detector. This description has entered the literature as the hot spot model (*15*) and it is also corroborated in our experiments below.

SNWDs were first developed for applications in photonics (*15*) where response times below 20 ps (*18*, *19*) are possible. Detection efficiencies of up to 99.5% can be realized even at telecom wavelengths (*20*). In addition, single photon sensitivity in the mid-infrared range of 10 μm wavelength has been demonstrated (*21*). SSPDs are impact detectors but were also designed to have photon number resolution (*22*). They have found applications in the explorations of the foundations of physics (*23*, *24*), quantum optics (*25*, *26*), and quantum information processing (*20*, *27*), astronomy (*28*), and molecular science (*29*). Recently, nanowire detectors have been demonstrated in TOF-MS (*16*, *17*, *30*, *31*) and more recently down to the kilo–electron volt level (*32*) and even for low-energy neutral molecules, in that case however, without unambiguous mass identification (*33*). Here, we explore high-resolution quadrupole mass spectrometry of proteins in low charge states with highly efficient SNWD arrays, at energies well below the requirements of conventional secondary electron detectors. In an effort to explore how to scale this technology up, we study different detector sizes and geometries and explore low-noise cryogenic electronics that can enable large multi-pixel arrays.

## RESULTS

### QMS-SSPD mass spectrometer

**Fig. 1.**
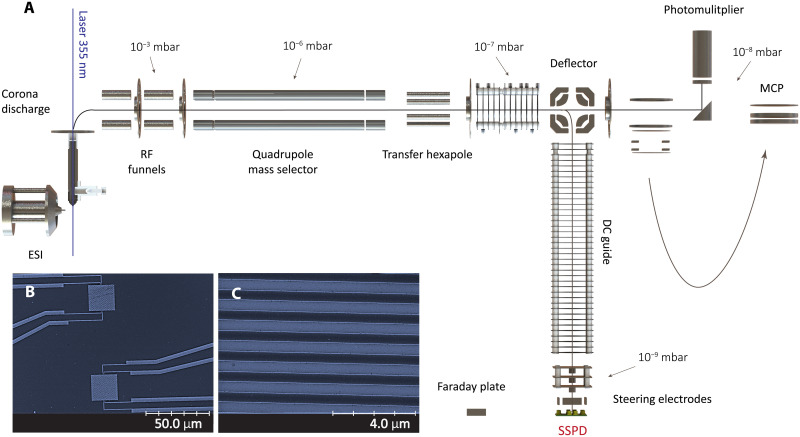
Quadrupole mass spectrometry with superconducting single particle detection. (**A**) Proteins are volatilized using electrospray ionization charge-reduced in bipolar air by a corona discharge. Further charge reduction by photochemistry is enabled by a high-power ultrafast ultraviolet laser interacting with the molecules in the entrance chamber. The ions are filtered in a quadrupole mass selector and pass through a radio frequency hexapole guide toward a quadrupole deflector. The ions are then steered to either a TOF-MS with multichannel plate (MCP), a phosphor screen with photo-multilpier (PhS), or the SSPD array. A Faraday plate can be shifted into the position of the SSPD to calibrate the incident ion current at high flux. Panel (**B**) shows an electron micrograph of two pixels (SSPDs), each with a size of 20 × 20 μm^2^ (**D_1_**), while (**C**) shows a close-up image of a detector pixel (**D_2_**) that has a 100 times larger area and a 500-nm line width, as also described in the text.

We have customized a TOF-MS, with an electrospray ionization source (ESI), extending it by adding corona charge reduction and a photo-cleavage stage, as well as an electrostatic ion switch that allows steering the ions after the quadrupole mass filter to either a conventional TOF-MS or toward the SSPD in a dedicated cryogenic vacuum chamber (Fig. 1). Proteins and other test molecules in our experiments are first individualized by electrospray ionization (*34*) and then optionally charge reduced in a corona discharge (*35*). The ions enter from ambient pressure through a 0.5-mm open cone into a pre-vacuum chamber at about 1 mbar before being guided by static ring electrodes into a quadrupole mass selector at <10^−6^ mbar. For photochemistry experiments, we have enabled an optical channel in this chamber to allow for a picosecond laser beam (355-nm wavelength, 500-kHz repetition rate, 90-μJ pulse energy, 0.5-mm beam diameter) to be focused through the source cone and exiting through a back window.

The quadrupole is customized to cover an exceptionally high mass-to-charge ratio of *m/z* = 30,000 Da/e, with a resolution of *Δm*/*m* ≃ 1:1000. A subsequent hexapole ion guide can serve to explore collision-induced dissociation or to implement buffer gas cooling. In our present experiments, it solely serves as an ion guide. In the original design of the machine, the ions are steered toward a TOF-MS followed by a multichannel plate (MCP) detector. Alternatively, the ions can be directed onto a phosphor screen and photomultiplier (PhS) to benchmark this established detector technology with our SSPD detector. We designate these three different detection modes as TOF-MCP, QMS-PhS, and QMS-SSPD mass spectrometry. To realize the QMS-SSPD mode, we have added an additional vacuum chamber between the QMS and the TOF filter. It contains a static quadrupole deflector that can bend the ions into a set of static ring electrodes, which guide the proteins toward a differentially pumped vacuum chamber where they can be focused by a set of Einzel lenses and *x*/*y*-deflected by two sets of plane-parallel electrodes. The ion beam is aimed at the superconducting detector, which is mounted inside a double-layer gold-plated copper shield to be protected from thermal radiation. The outer shield is thermally anchored to the 40-K stage, while the inner shield is anchored to the 3.7-K stage of a pulse tube cooler (for details, see Materials and Methods). An additional Faraday detector can be inserted into the ion beam line for the determination of the total ion current.

### Superconducting nanowire detector

We have studied three different detector types, all manufactured as arrays of 9.5-nm-thick NbTiN nanowires with a critical temperature of *T_c_* ≃ 10 K (*36*). Detector **D**_**1**_ fills an area *A*_1_ of 20 × 20 μm^2^ in meander form with a line width *w*_1_ of 100 nm and a pitch *p*_1_ of 200 nm, i.e., with a fill factor of 50%. To increase the detection area, 32 pixels of **D**_**1**_ were combined in a linear array, of which every pixel would have to be connected by individual leads to the signal conditioning and processing electronics at room temperature. Parallelization of elements leads to high thermal load and suggests the use of cryogenic onboard electronics. In preparation for that, we have realized an array of eight pixels of type **D**_**1**_, where one low-noise amplifier (LNA) has been added to every pixel on board, which will be referred to as chipset **D**_**1b**_. For beams of high flux, the high spatial resolution of detector **D**_**1**_ is appealing. Since this detector has a small kinetic inductance, it also offers faster relaxation times and saturates at high molecular flux. However, in molecular beam research and mass spectrometry, total detector size is key. Therefore, we have increased the individual pixel area by a factor of one hundred to *A*_2_ = 200 × 200 μm^2^. Our detector **D**_**2**_ combines 10 such pixels, each with a line width *w*_2_ = 500 nm and pitch *p*_2_ = 1000 nm, each individual element exceeding the detection area of a full 32-pixel chip of **D**_**1/b**_ by three times. Six of the 10 pixels of detector **D**_**2**_ were connected to room-temperature electronics.

The two detector geometries differ not only by 100 times in area and counts but also critically in their dark count rate. Detector **D**_**1**_ has 100-nm-wide wires with low heat capacity. It is sensitive to photons and must be shielded from stray radiation when operated close to the switching current. In contrast to that **D**_**2**_ is blind to black body radiation and achieves a dark count rate as low as 0.02 cps under normal operating conditions. Since we operate at low molecular flux, our progress toward realizing high pixel numbers with **D**_**1**_ is shown in the Supplementary Materials, while all data in the main text are recorded with either a single pixel or six pixels of detector array **D**_**2.**_

### Demonstration and benefits of QMS-SSPD mass spectrometry

In this section, we show the application of the SSPDs in continuous quadrupole mass spectrometry. We show that even lowly charged high-mass proteins can be well detected and that the QMS-SSPD spectrum has a good mass resolution and a good signal-to-noise ratio. This method can distinguish different impact energies and, as a result, discriminate different charge-to-mass states, which is typically not possible in QMS or TOF mass spectrometry. We show that molecular momentum and molecular composition do not influence the detection in the observed mass and energy range, ensuring a large range of applications for SSPD devices.

### QMS-SSPD protein mass spectra at low-impact energy

**Fig. 2.**
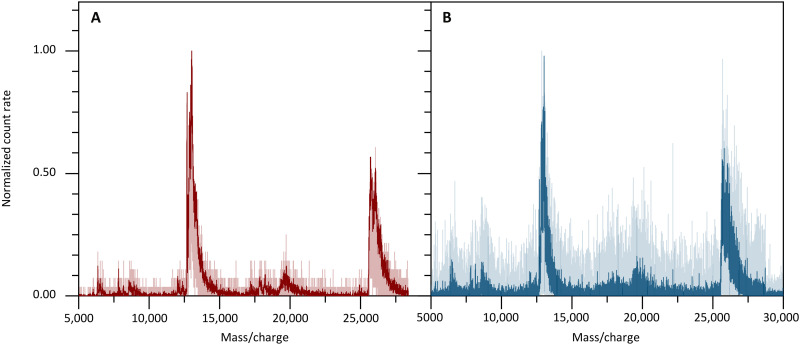
Impact energy in QMS-SSPD versus QMS-PhS mass spectra. (**A**) Charge-reduced concanavalin A recorded by the SSPD (**D_2_**) at 190 V and (**B**) a phosphor screen detector at 10 kV. Panels (A) and (B) show solid lines representing data smoothed using 15-point and 25-point moving averages, respectively.

In the first set of experiments, shown in Fig. 2, we compare the mass spectrum of charge-reduced concanavalin A, as seen by the nanowire detector (Fig. 2A) and as recorded by the phosphor screen (Fig. 2B). Both recordings start from electrospray ionization of proteins, corona charge reduction, and quadrupole mass filtering. Both mass spectra show good signal-to-noise and identical mass peaks, but this at vastly different ion impact energy.

At each detector, the kinetic energy *E*_kin_ = *q* · *U*_acc_ is determined by the charge *q* of the ions and by the acceleration potential *U*_acc_, which amounts to *U*_acc_ = 10 kV at the phosphor screen and to 13 kV at the MCP behind the TOF-MS. In contrast to that, the superconducting detector can measure a mass spectrum at *U*_acc_ ≃ 190 V and it still works for impact energies down to 50 eV (see below), i.e., it accepts 50 to 200 times lower impact energies than required by electron multiplying devices.

The fact that QMS-SSPD mass spectrometry can acquire data continuously is used to demonstrate photo-uncaging of individualized photo-active tags, interacting with a fast-pulse ultraviolet laser beam in the gas phase at high repetition rate. Upon absorption of one or two photons, the leaving group is detached, and with it the charge that is located on the tag (see Supplementary Materials). We show a proof-of-concept photo-cleavage experiment using QMS-SSPD mass spectrometry in the Supplementary Materials (fig. S9).

**Fig. 3.**
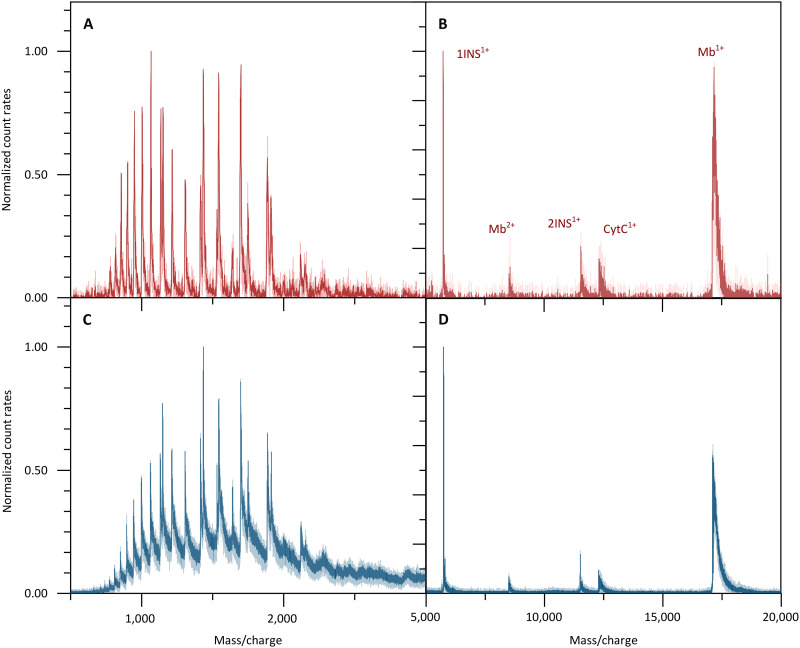
Signal background and complexity in QMS-SSPD versus TOF-MCP spectra. The sample is a mixture of insulin, cytochrome C, and myoglobin. The signal background of QMS-SSPD with detector **D_2_** (**A**) is lower than that of TOF-MS (**C**). Charge reduction facilitates the peak assignment and QMS-SSPD detection shows higher counts at higher mass to charge states (**B**) than the TOF-MS MCP detector (**D**). The solid lines represent a five-point moving average of the data. Note that while the spectra in both detector modes resemble each other, as they should, the QMS-SSPD data were recorded at 50 times lower kinetic energy of the ions.

If more than a single charge state needs to be removed, a corona discharge in the air allows us to study proteins in reduced charge states. This simplifies the mass analysis, and in combination with the SSPD detector, it allows the detection of higher masses with higher sensitivity than conventional detectors. This is shown in Fig. 3, where we display the mass spectrum of a protein mix containing insulin, cytochrome C, and myoglobin. They were recorded using the QMS-SSPD (Fig. 3, A and B) and the TOF-MCP (Fig. 3, C and D) detector. The electrospray displays proteins in high charge states, which are well recorded by both detectors.

Corona charge reduction facilitates the mass assignment enormously as only singly and doubly charged proteins appear. The spectrum reveals that the superconducting detector Fig. 3B surpasses the commercial MCP detector (Fig. 3D) at high masses: It finds singly charged myoglobin with a high relative count rate even when operated at more than 200 times lower-impact energy. Note that while this qualitative trend is in full agreement with our expectations, differences in transmission through the ion guides may also play a role.

All recordings of Fig. 3 were acquired with six pixels of array **D**_**2**_ combined into a single superpixel. This increases the effective detector area to 0.24 mm^2^, compared to an ion beam area of about 3 mm^2^. The recordings were taken with an integration time of 1200 s (Fig. 3, A and B) in QMS-SSPD mode, while the spectra using TOF-MCP (Fig. 3, C and D) required 120 s because of the larger detector area. Notably, while electron multiplying detectors can be built in sizes beyond a square inch, this is mostly a matter of convenience, as typical ion beams can be substantially smaller than that (see below).

### Dependence on molecular energy, charge, momentum, and structure

Having seen that SSPDs are excellent molecular impact counters for quadrupole mass spectrometry, we now analyze how molecular impact energy, charge, momentum, and structure influence the detector signal and how the signals align with an established model.

### Energy and charge dependence

**Fig. 4.**
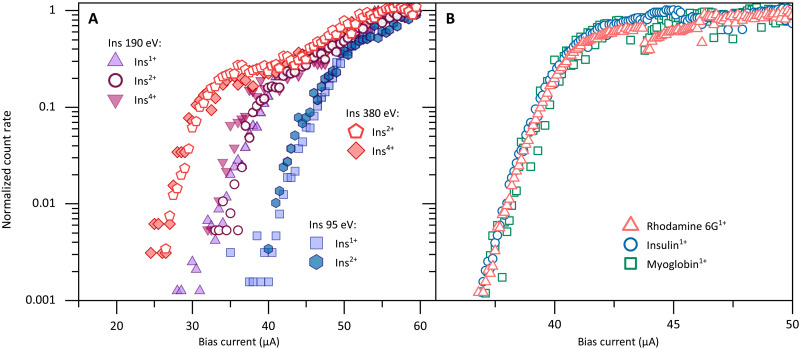
Influence of energy/mass/momentum/structure on the detection mechanism. (**A**) Energy dependence of the normalized count rate. The normalized protein count rate is shown as a function of the SSPD (**D_2_**) bias current *I_b_*, for insulin in the charge states *q = +1e*, *+2e*, and *+4e* and for the acceleration voltages *U*_acc_ = 47.5,95 V,142.5,190 V. The observation of three groups of data confirms that the detection efficiency depends not only on the bias current but also on the kinetic energy *E*_kin_ = *q* · *U*_acc_, but not separately on the charge of the molecule. This can enhance standard mass spectrometry, where *m/z* is the prime quantity. (**B**) Mass/momentum/structure independence. At equal impact energy of *E*_kin_ = 190 eV and equal charge, we find the same normalized detector response curve for molecules of vastly different structure and complexity, specifically rhodamine 6G, insulin, and myoglobin. This suggests that kinetic energy is of prime relevance, while molecular structure, atom number, charge, and mass are not.

In Fig. 4A, we plot the insulin ion count rate as a function of the detector bias current *I*_b_ for a single pixel of **D**_**2**_. The curves are taken for varying molecular charge states *q* and different values of the acceleration voltage *U*_acc_. We find that ions of the same kinetic energy *E*_kin_ fall onto the same curve, while ions at different kinetic energies (95, 190, and 380 eV) can be clearly discerned by their onset bias current *I*_on_, which we define as the intersection of the extrapolated ion signal with the *x* axis. Figure 4A suggests that under our experimental conditions, an energy resolution of 20 to 30 eV can be achieved, corresponding to a difference in the onset bias current of Δ*I*_on_ = 1 μA. This also shows that ions of the same mass-to-charge ratio, but different charges, will be distinguishable when monitoring the SSPD bias current curves in addition to the QMS transmission. An example of that would be the comparison of a doubly charged insulin dimer with a singly charged insulin monomer accelerated by the same potential *U*_acc_.

### Momentum dependence

For particles to create a normal-conducting hot spot, they must couple to either the charge carriers or the phonons in the nanowire. It is therefore interesting to explore if it is possible to distinguish between the two effects. Since for fixed kinetic energy, the momentum transfer increases with mass, *p* = (2*mE*_kin_)^1/2^, we study the bias current curves for molecules of the same kinetic energy, *E*_kin_ = 190 eV, but different mass, momentum, and number of constituents. Figure 4B shows count rates for singly charged rhodamine 6G (C_28_H_31_N_2_O_3_Cl, *N* = 65, m = 479 Da), bovine insulin (C_254_H_377_N_65_O_75_S_6_, *N* = 777, m = 5733 Da), and myoglobin (153–amino acid residues, *N* = 1304, m = 16952 Da). They cover a mass ratio of about 1:12:35, a momentum ratio of about 1:3.5:6, and a ratio in the number of atoms of about 1:12:20.

While we cannot compare their absolute detection efficiencies because of potentially different transmission through the ion guides, we find that the onset bias current *I*_on_ only depends on energy, but not on molecular mass, momentum, or structure—under our experimental conditions. This is interesting as one might expect to see the influence of molecular deformation or dissociation during impact as possible energy loss channels which would depend on atom number and complexity. Future modifications of the ion guides shall enable the exploration of that mechanism at ion energies down to 1 eV. The observed composition independence for energies above 100 eV is favorable for mass spectrometry, and it is a feature that we exploit below in our calibration of the total quantum yield.

### Confirming the hot spot model

**Fig. 5.**
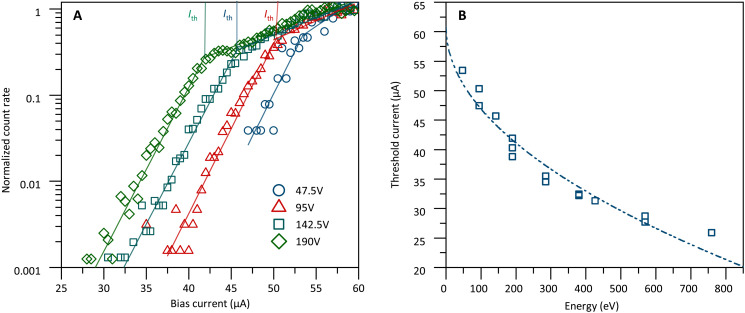
Confirmation of the hot spot model for detector **D_2_**. (**A**) Threshold currents: From the bias current scans a threshold current *I*_th_ can be defined by finding the intersection of the asymptotes (straight lines) of the count rate as a function of the bias current *I*_b_. (**B**) Hot spot detection model. When we plot *I*_th_ for insulin as a function of kinetic energy, the data are well fitted by the hot spot model (dashed line; see text).

In all curves shown in Fig. 4, a threshold bias current *I*_th_ can be defined as the intersection of the asymptotes at low and high bias currents (*37*). This is shown in Fig. 5A and the dependence of *I*_th_ on the molecular impact energy is shown in Fig. 5B. It is well reproduced by the analytical form $I_{\mathrm{th}}=I_{c}(1-\gamma \sqrt{E_{\mathrm{kin}}}/w_{2})$, which is predicted by a normal-core hot spot model (*32*).

Here, *w*_2_ = 500 nm is the width, *I*_c_ is the critical current of the nanowire, and the parameter γ is governed by the density of states at the Fermi level as well as the efficiency of quasiparticle creation. After fitting the data, we find that γ =1.2 × 10^−8^ eV^−1/2^m and *I*_c_ = 61 μA. We can extract important information about the transduction mechanism by comparing *I*_c_ with the depairing current *I*_dep_(*T*) = *I*_dep_(0)[1 – (*T*/*T_c_* )^2^]^3/2^.

Here, *I*_dep_(0) = 0.74 *w*_2_ Δ(0)^3/2^/(*eR*_□_*ℏD*) and Δ(0) are the depairing current and superconducting energy gap at *T* = 0 K. With electron charge *e*, the critical temperature *T*_c_ = 10.5 K, the sheet resistance *R_□_ = * 380 ohms, and the electron diffusion coefficient *D =* 0.48 cm^2^/s (*38*) specifically for NbTiN (*39*), we obtain *I*_dep_(0) ≃ 350 μA and *I*_dep_(3.7 K) ≃ 285 μA at our working temperature.

We find *I*_c_/*I*_dep_ = 0.2, which is consistent with a hot spot detection model (*15*) and clearly distinct from a vortex-assisted detection model (*40*) which requires *I*_c_/*I*_dep_ > 0.5. In addition, we estimate the hot spot diameter for our lowest ion impact energies (50 eV) to be $D_{HS}=4\sqrt{\tau_{\mathrm{th}}\;D}≃35$ nm, where τ_th_ ≃ 1.7 ps is the thermalization time.

### Protein ion beam profiling

**Fig. 6.**
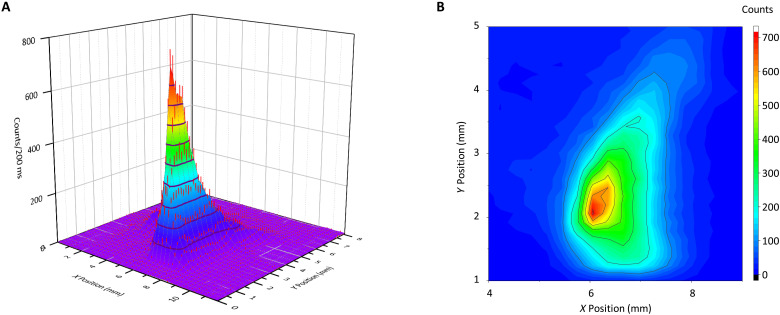
SSPD beam profile of a mass-selected Insulin (Ins^5+^) ion beam at 1000 eV of impact energy. To illustrate the signal-to-noise ratio and the beam shape, the same data are shown as a three-dimensional image (**A**) and as a contour plot (**B**). The *X* position represents the mechanical shift of one SSPD pixel (**D_2_**). The *Y* shift quantifies the mechanical shift of the ion beam generated by the deflection in an external field (see the Supplementary Materials). The asymmetry of the ion beam is due to the geometry of the ion bender.

Given the high sensitivity and good resolution of superconducting detectors for massive particles, we proceed to record a spatial image of a protein ion beam. This will also be used to derive the total quantum yield for detector **D**_**2**_. Figure 6 shows the measured beam profile of insulin^+5^ with an impact energy of 1 keV that is sufficiently well-resolved to monitor the ion beam steering. A pair of *X*/*Y* electrodes in front of the SSPD detector are used to deflect the incident ion beam with high reproducibility. They are calibrated with respect to a mechanical translation stage, with 10-μm accuracy.

### Total detection quantum yield

SSPDs are known to be efficient photon detectors, but varying numbers have been reported in the literature on the total quantum yield for low-energy ions (*41*). Also, the absolute calibration for molecules at low kinetic energy had been an open challenge. Normalized count rates as shown in Fig. 4 may lead one to assume that observing saturation is an indicator for an area-normalized quantum yield close to 100%. However, this could be wrong for fast atomic ions, which can penetrate the nanowire and thus deposit only a fraction of their energy. It could also be wrong for fast proteins if they convert their impact energy into internal vibrational excitation or even dissociation.

**Fig. 7.**
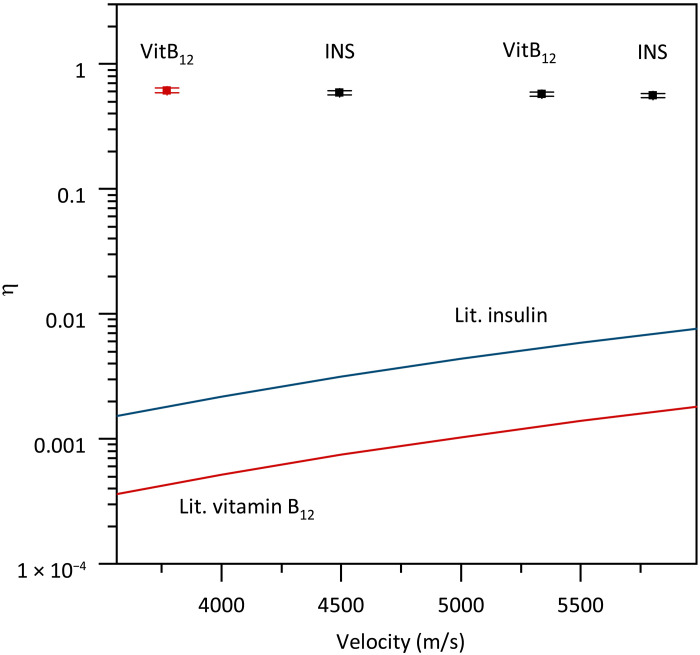
Total molecule detection quantum yield. The black squares show the average of the total detection quantum yield of two different pixels of detector **D_2_** as a function of impact velocity for (from left to right) insulin at 600 eV, vitamin B_12_ at 200 eV, and insulin at 1000 eV of impact energy. The red data point for vitamin B_12_ at 100 eV (left) was recorded using six individual pixels of the second detector array **D_2_**. They all show the same total quantum yield within the error bar. The red and blue lines show expected literature values for MCPs (*42*). For a detailed discussion of the quantum yield and its uncertainties, see the Supplementary Materials.

To solve this question quantitatively, we here relate the single-molecule count rate of two separate pixels of **D**_**2**_ to the electrical current that is measured by a large Faraday plate that can be inserted into the ion beam. The total quantum yield was measured for singly charged vitamin B_12_, insulin^5+^, and insulin^3+^ for two different impact velocities (energies). Mixing the molecular species and their charge states is justified since our measurements above have shown that the detection process is independent of molecular mass and structure in the given energy range. Low-mass ions are favored for this test because of their higher flux, which facilitates comparing the SSPD signal with that seen by a femto-ammeter. The individual ion beam profiles compare well with those recorded in Fig. 6 and are used to account for the size differences between the SSPD detector (200 × 200 μm^2^) the ion beam (about 2 × 3 mm^2^ with asymmetric shape) and the Faraday plate (1 × 1 cm^2^). By integrating the signal of all SSPD profile points and comparing them to the total ion current seen by the Faraday plate, we derive the total molecule detection quantum yield. We have done this for insulin as well as vitamin B_12_ at various energies in the range of 100 to 1000 eV, as shown in Fig. 7. We obtain a total detection yield of η = 0.62 ± 0.03 which even corresponds to a nominal area–normalized quantum yield of 124% when we only consider the nominal surface filling factor of the nanowire meander of 50% (for a discussion, see the Supplementary Materials). The observed total detection yield is remarkable and consistent among all detectors. It exceeds those of MCPs (*42*) by between two and more than three orders of magnitude for ions of identical impact velocities.

## DISCUSSION

We have demonstrated that superconducting nanowire arrays can serve as highly efficient detectors for massive ions even at energies where conventional detectors fail. We can record mass spectra of singly charged insulin down to kinetic energies 50 to 100 times lower than typically required for TOF-MS. This is compatible with and beneficial for continuous mass spectrometers, such as quadrupole or sector field instruments, where mass resolution even improves with decreasing ion energy.

The energy dependence of the SSPD threshold current *I*_th_ makes distinguishing *n*-fold charged polymers of a given mass possible, whereas conventional detection methods can only infer the presence of isobaric species of different charges by analysis of the isotope distribution. This can be best observed at low ion energy, where the rate of increase for the threshold current *I*_th_ is greatest.

We have demonstrated a total quantum yield of η = 0.62 and an area-normalized quantum yield slightly above 100% for molecules with kinetic energies of 100 eV or higher, independent of their structure, mass, charge, or momentum.

At lower energy, the yield may be influenced by molecular mass and complexity since molecular dynamics simulations show that soft matter can deform, fragment, or bounce off after surface impact (*43*, *44*). Although such excitations will eventually relax to the nanowire, this process occurs on a timescale of nanoseconds, much slower than the relaxation time of the superconducting detector. Also, the coating of the detector by the protein beam or residual gas can act as an energy-dissipating buffer for succeeding particles. However, to deposit a single monolayer of proteins on the detector surface, one would need to run the mass spectrometer continuously for a few hundred hours under normal conditions and organic debris can be easily cleaned off. In addition, at a base pressure of 10^−9^ mbar, a gas cushion is not observed.

While mass spectrometry and neutral molecular beam research would already profit from a 10 × 10 array of type **D_2_**, more versatile applications with even better spatial resolution will emerge when it becomes possible to scale the smaller pixels of array **D_1_** to substantially higher numbers. Since individual cabling to room temperature becomes excessive in space requirements and heat dissipation for more than about 100 pixels, we here analyze some thermal limits related to cryogenic onboard current sourcing, signal amplification, and readout. Although superconducting nanowires transport current without loss, the bias current *I*_b_ is generated by sending a voltage into a resistor. For small detectors like **D_1/b_**, the power per pixel can be individually set and limited to *P* = 36 nW if we assume a bias current of *I*_b_ ≃ 6 μA and a source resistance of *R* = 1 kilohm.

Our experiments with detector **D_2_** have shown that the sensor area can be increased by a factor of 100 when the bias current is increased by a factor of 10 and the power by 100 times. The same cooling power budget would thus allow sourcing all these detectors individually across a total detector area beyond 40 cm^2^. The true challenge comes on the amplification side.

To tackle this challenge in a proof-of-principle experiment, we have developed an array of eight onboard cryogenic LNAs in SiGe and integrated them into our QMS-SSPD mass spectrometer. They consume less than 6 mW per pixel, allowing them to realize dozens to 100 individually amplified pixels using analog electronics at 4 K. The detector design is illustrated in fig. S6. It is embedded in the experiment in fig. S7.

The full power of this idea will be harvested when hundreds of these detectors can be operated in parallel. Also, signal multiplexing at high clock frequency could open a way to sampling more than 100 amplified signals, thus pushing the detector size to more than 1000 individually controlled pixels. Such large, well-resolving devices with high total detection quantum yield and high control over each individual pixel will become important for atomic and molecular beam research with low-energy particles as well as for continuous mass spectrometry with lowly charged high-mass molecules.

## MATERIALS AND METHODS

### Experimental design

#### Customized mass spectrometry

A Waters Q-TOF Ultima mass spectrometer was customized. (i) The RF electronics of the quadrupole mass filter were upgraded by MSVision for a molecular mass-to-charge ratio of *m/z* = 30 kDa/e. (ii) A differentially pumped vacuum chamber was inserted between the quadrupole mass filter and the TOF-MS to add a static quadrupole bender, static ring ion guides. (C) A differentially pumped cryogenically cold ultrahigh vacuum chamber hosts the nanowire detectors and a Faraday plate. The cryostat is mounted on a motorized translation stage that can vertically move the SSPD with 10-μm accuracy. The detectors are cooled by a Sumitomo pulse tube cooler (RH), with a nominal cooling power of 900 mW at 4.2 K. The system is usually operated at 3.7 K. (iv) The mass spectrometer entrance region was modified for corona charge reduction and laser-induced photo-cleavage.

#### Nanowire fabrication

A 180-nm-thick SiO_2_ layer is grown on a Si wafer. The 9.5-nm superconducting thin film is then deposited by reactive magnetron co-sputtering of Nb and Ti in a dilute Ar^+^ and N_2_ plasma. After the NbTiN layer is deposited, the nanowire meander structure is written by electron beam lithography using an E-beam resist. The pattern is then transferred to the NbTiN film by reactive ion etching with SF_6_ and O_2_. As a final step, the E-beam resist is removed, and the nanowire meander is exposed.

## Supplementary Materials

### This PDF file includes:

Supplementary Text Figs. S1 to S13 Table S1 References
