# Supplementary material for: Highly sensitive single-molecule detection of macromolecule ion beams
Source: Sci Adv. 2023 Dec 1;9(48):eadj2801. doi: 10.1126/sciadv.adj2801 (PMC10691769; doi:10.1126/sciadv.adj2801)
Supplement: Supplementary file 1 — Supplementary Text Figs. S1 to S13 Table S1 References [file sciadv.adj2801_sm.pdf]

Science Advances
AAAS

Supplementary Materials for

Highly sensitive single-molecule detection of macromolecule ion beams

Marcel Strauß *et al.*

Corresponding author: Markus Arndt, markus.arndt@univie.ac.at

Sci. Adv. **9**, eadj2801 (2023)
DOI: 10.1126/sciadv.adj2801

This PDF file includes:

Supplementary Text
Figs. S1 to S13
Table S1
References

1. Mass spectrometry

Sample preparation and beam source

The following dyes, vitamins and proteins were purchased from Merck and prepared with concentrations, purity and solvents as listed in **Table S1**:

Molecule	R6G	Vit B_{12}	INS	CytC	Mb	Con A
MW	0.44 kDa	1.58 kDa	5.73 kDa	12.32 kDa	17.74 kDa	25.72 kDa
Conc.	200 μM	20 μM	20 μM	20 μM	20 μM	20 μM
Purity	99%	99%	> 25 USP units /mg	> 95%	>95	> 90
Solvent	C_2H_3N / H_2O (1:1)	H_2O	H_2O	H_2O	H_2O	C_2H_3N / H_2O (1:1)

Table S1: Analytes and chemical parameters used in the detector experiments.
Vitamin B12 (Vit B_{12}), rhodamine 6G (R6G), insulin bovine pancreas (INS), cytochrome C from bovine heart (CytC), myoglobin from equine skeletal muscle (Mb) and concanavalin A (Con A).

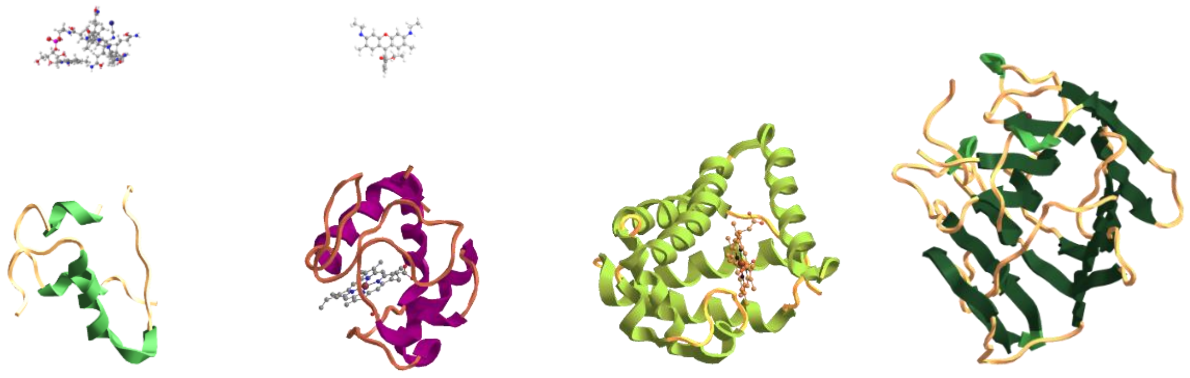

Figure S1: Gallery of molecules used in the experiments.
The shapes are meant to provide a comparison of size and complexity, but do not indicate the molecular conformation in transit through the mass spectrometer or during impact on the detector.

All molecules were electro-sprayed using an 80 μm needle operated at 3 to 4 kV. The solution is driven by a Harvard syringe pump at 9 μl/min. Optionally, the molecular charge is reduced in a corona discharge for which nitrogen 5.0 is passed through pure ethanol at elevated temperature and sent into a chamber with about 1 cm^3 volume. A sharp stainless steel needled at -3 kV induces the discharge which is actively stabilized at a current of 15 μA. The resulting bipolar air is guided into an aluminum channel of 3 mm inner diameter and 80 mm length where it mixes with the electrospray before entering the vacuum system of the mass spectrometer.

Data acquisition and mass calibration

An QMS-SSPD mass spectrum is recorded. A programmable pulse generator (PPG) triggers a scan of the quadrupole mass spectrometer, while the time to digital converter (TDC) collects the SSPD ion counts. Since the QMS amplitude scan is linear, the SSPD spectrum can be referenced to a calibrated spectrum, here recorded in situ using TOF-MS.
For calibration purposes we can alternatively steer the ion beam in the forward direction, where it is either detected using a conversion dynode at -10 kV, a phosphor screen and a photomultiplier

(QMS-PhS) or using a time-of-flight mass spectrometer with MCP detector at -13 kV (TOF-MCP). The TOF-MS spectrum is first calibrated using cesium iodide clusters and then used to calibrate the QMS-SSPD system, as shown in **Figure S2**, for a mixture of cytochrome C, insulin, and myoglobin.

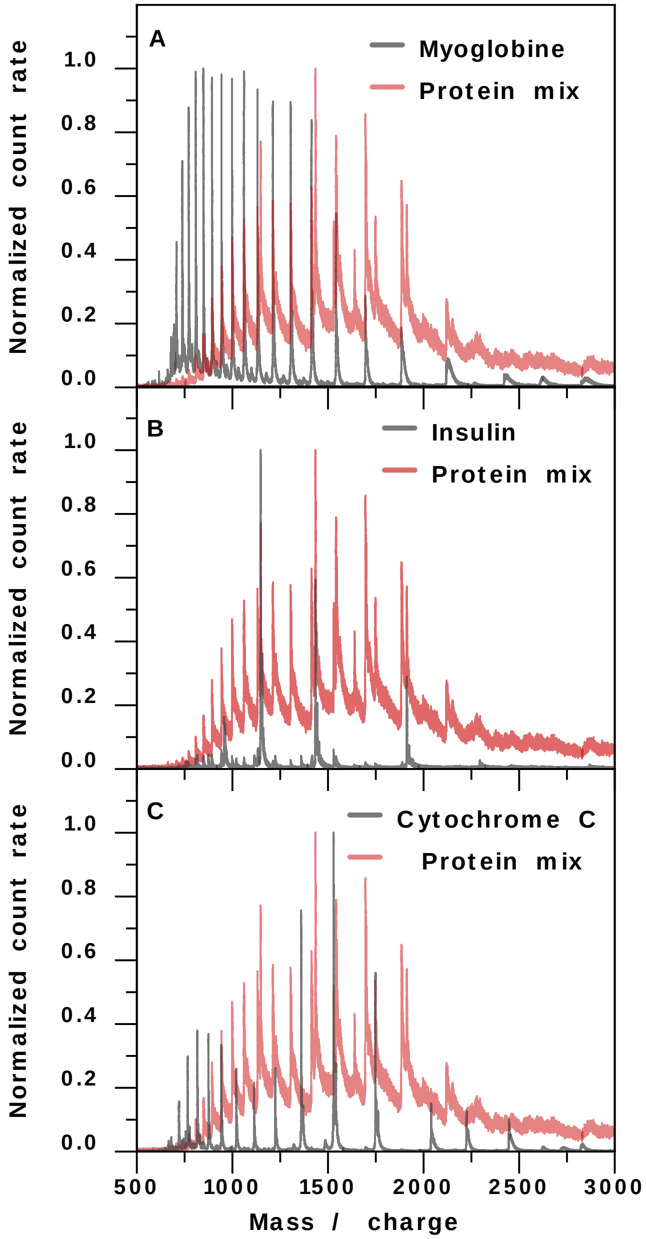

Figure S2: Identification of protein mass spectra.
The mass spectrum of a protein mix of myoglobin (A), insulin (B) and cytochrome C (C) is compared with the mass spectra of the individual proteins using a calibrated TOF-MS. The peaks are used to calibrate the QMS-SSPD measurements, **Figure 3** in the main text.

2. QMS-SSPD mass spectrum recorded using detector D_1

In **Figure S3** we show an insulin mass spectrum recorded using detector **D_1**. While the system fulfills its function, the integrated surface of even an 8-pixel detector of **D_1** is still an order of magnitude smaller than a single pixel of detector **D_2**. This explains why all figures in the main text were recorded using the large area detectors. A new generation of experiments shall combine more than a hundred small or large detectors into versatile systems whose shape, resolution and area can be tailored to the needs of the experiment.

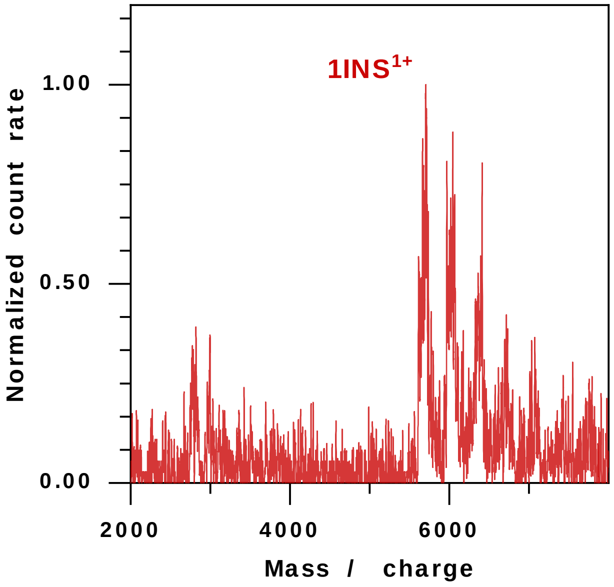

Figure S3: QMS-SSPD spectrum measured with detector D_1.
Mass spectrum for Insulin^{1+} measured with 2 pixels of chipset **D_1**. The working principle is clearly corroborated. For mass spectrometry and molecular analysis, integration of hundreds of these small pixels will become important to improve detector area and signal-to-noise. The additional side peaks are due to adducts generated in the ESI source region. The data was smoothed using a 25-point moving average.

3. Superconducting detector

The detector assembly is bolted onto the cold head of a pulse tube cooler (Sumitomo SHI R65p) which can be cooled to 3.7 K (nominal 900 mW at 4.2 K) with all conditioning and signal cables connected. The SSPD detector is shielded by two gold coated OFHC copper cylinders with a 10 mm entrance hole for molecules and light. Visible photons can be shielded by covering all windows and their detection can be suppressed by choosing the bias current appropriately.

Note, however, that the small detectors **$D_{1(/b)}$** are sensitive to single visible and near-infrared photons, and will therefore always see some background, while the larger wires of **D_2** require slightly higher energy to trigger a hot-spot and can be operated with dark count rates as low as 0.02 cps.

The superconducting nanowire detectors are connected to a constant current source (Single Quantum), which drives each detector pixel separately with 6 to 20 µA for detector **$D_{1(/b)}$** or 10 to 65 µA for detector **D_2**. The signal after ion impact is guided via a bias-T to two subsequent 20 dB voltage amplifiers (Mini circuits), followed by the TDC. The cryogenic assembly is mounted on a motorized translation stage to either mechanically scan the molecular beam profile or to lift the Faraday detector into the beam.

4. Calibration of the ion beam profiler

The ion beam profile of **Figure 6** in the main text was recorded using detector **D_2**. In the vertical direction, the profile can be scanned either by mechanically shifting the detector or by tuning the voltage of the ion deflector. Since the horizontal and vertical deflectors have identical geometries, the vertical calibration of the electrodes is also used for the horizontal axis. During the calibration process the deflector voltage was increased in steps of 10 V or 20 V, while the nanowire detector was mechanically moved in steps of 200 µm, with 10 µm accuracy over a distance of 10 mm, collecting a slice of the ion beam profile.

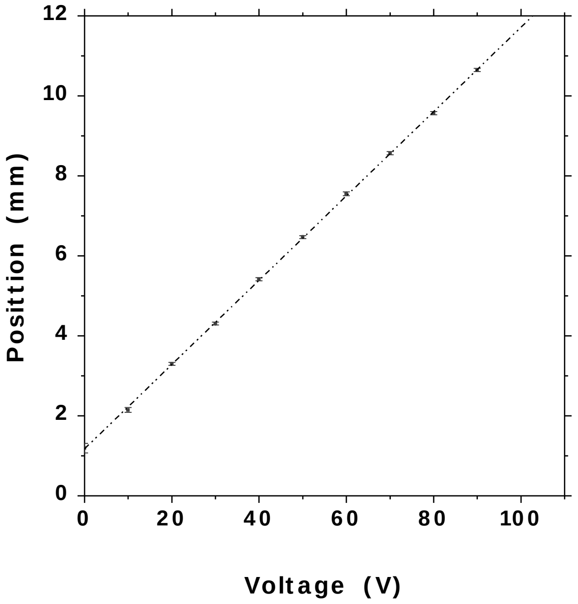

Figure S4: Calibration of the SSPD ion beam deflector (1000 eV insulin).
The deflector calibration curve represents the barycenter and the uncertainties of a fit to the ion beam profile as a function of voltage applied to the electrode. The high linearity allows for an accurate representation of the ion beam profile in **Figure 6** of the main text.

The mean and standard deviation of the center position of these slices are then determined and plotted against the deflector voltage, along with an upper bound for the error due to inaccuracies in the mechanical position of the SSPD (10 µm). The result plotted in **Figure S4** shows a highly linear position-voltage dependence. The deflection voltage steps are chosen to produce a shift equivalent to the pixel-length of the detector (200 µm).

5. Systematic uncertainties in the total detection yield

The total detection yield of our system was experimentally determined to be $\eta = 0.62$. If we assume a geometrical filling factor of 50%, based on an evaluation of the electron micrographs, we deduce an area-normalized detection yield of even 124%. We here address how to understand and further analyze this observation:

Detector geometry

- If the meander filling factor is larger than the specified 50% this will not modify the experimentally determined total quantum yield, but it will increase the apparent area-normalized quantum yield. Our measurements indicate that the effective surface would be 20% larger than deduced from scanning electron micrographs, which nominally define the meander with an accuracy of 3% in one dimension.

- The macroscopic wires that are leading to the detector are thinned out from several micrometers down to the nanowire width of 500 nm in D_2. At 100 eV of impact energy even wider wire areas may still contribute to the signal. Systematic test using a series of different chip geometries will be required to elucidate this uncertainty.
- There can be contribution to the area-normalized detection yield from ions striking the SiO_2 substrate in proximity to the nanowires. This effect may be relevant considering that even at the lowest impact energy of 50 eV the hot-spot diameter is estimated to be 35 nm. If energy is released to the SiO_2 substrate and if that couples to the superconducting film, the effective nanowire width may be increased. The relevance of this effect requires a new series of experiments with nanowires of different "dead zones".
- We can finally convolute the finite diameter of a protein with the nanowire geometry. This results in an increased effective detector width. However, for insulin this effect is minimal, contributing less than 1% when the line width is 500 nm.

Source Stability

- If the protein flux varies during the normalization, this can change the total and area-normalized quantum yield. The flux was measured to be stable to within 3% over one hour.

Signal ringing

- For fast electronics, as typical in SSPD research, it is conceivable that cable reflections generate ringing, which may make that signals are counted more than once. The signals in our setting are optimized for mass spectrometry, using large area detectors, and we have not found indication for ringing in these measurements.

Ion beam profile

- To exclude the potential deformation of the ion beam during deflection, a 2d beam profile was measured at 0 V and 80 V deflection voltage by mechanically moving the detector along the same axis. This excludes any distortion of the ion beam by the deflection (**Figure S5**).

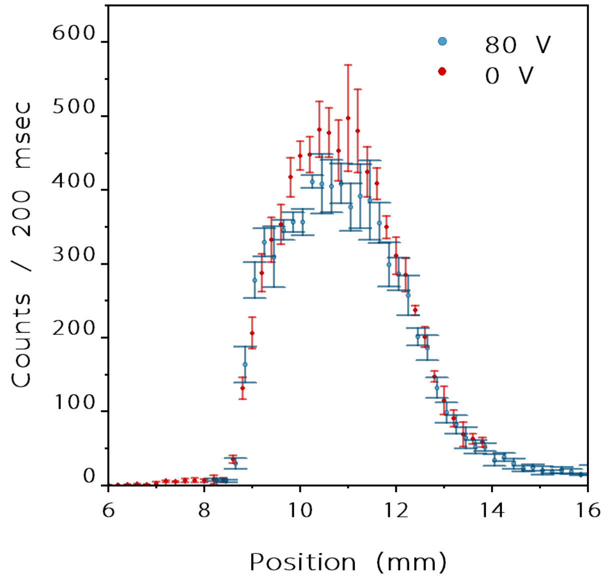

Figure S5: Beam profile at different deflection voltages.
The beam profile stays the same after deflection. However, the count rate changed a bit over time.

6. Cryogenic low-noise amplifier for scalable devices

With the goal to scale up the detector area and to improve the detector resolution, it is interesting to explore multi-pixel arrays and new ways to control their readout electronics in a cryogenic environment.

A large-area detector such as **D_2** is usually driven by a bias current of 30-50 μA which generates already a sizeable voltage pulse across a 50 Ohm impedance when the superconductor transitions to normal conductivity. However, the better resolving detector **D_1** is typically driven by < 6 μA and therefore generates only a 300 μV signal. Low-noise cryogenic amplifiers (LNA) are thus important to boost the signal-to-noise ratio and to reduce the rise time of the output pulse for future on-board digitization and time-tagging.

For that purpose, we have designed and realized a novel amplifier with high gain, high bandwidth, low noise, and low power consumption, using a heterojunction-bipolar transistor (HBT) in a BiCMOS process.

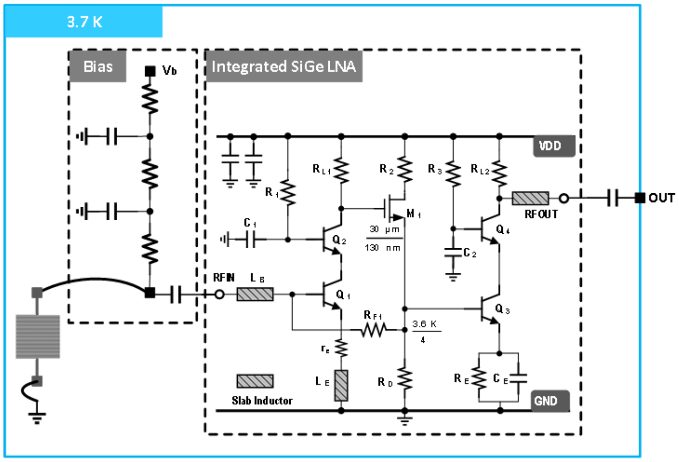

Figure S6: Cryogenic on-board low-noise amplifier.
Integration of a SiGe low-noise amplifier (LNA) with the superconducting nanowire detector on a shared printed circuit board at T = 3.8 K. These detectors with onboard electronics shall be used to integrate larger pixel numbers and larger detection areas.

The amplifier shown in **Figure S6** exhibits good noise and speed at room temperature and improves even its current gain by about 300 % upon cooling to T = 3.7 K (17,46,47) The device measures only $480 \times 280\ \mu m^2$ including the pads and it combines an average noise temperature of 5 K in the frequency range of 0.1 – 8.8 GHz. It has a good weak-signal linearity with well-matched input impedance (S11 < -10 dB) and a gain > 33 dB.

Integration of the LNAs into detector D_{1b}

To demonstrate this integrated concept, we have joined an array of 8 SSPD pixels of type **D_1** with eight of our new LNAs to realize eight detectors **D_{1b}** and embedded them into the cryogenic detector setup as illustrated in **Figure S7.**

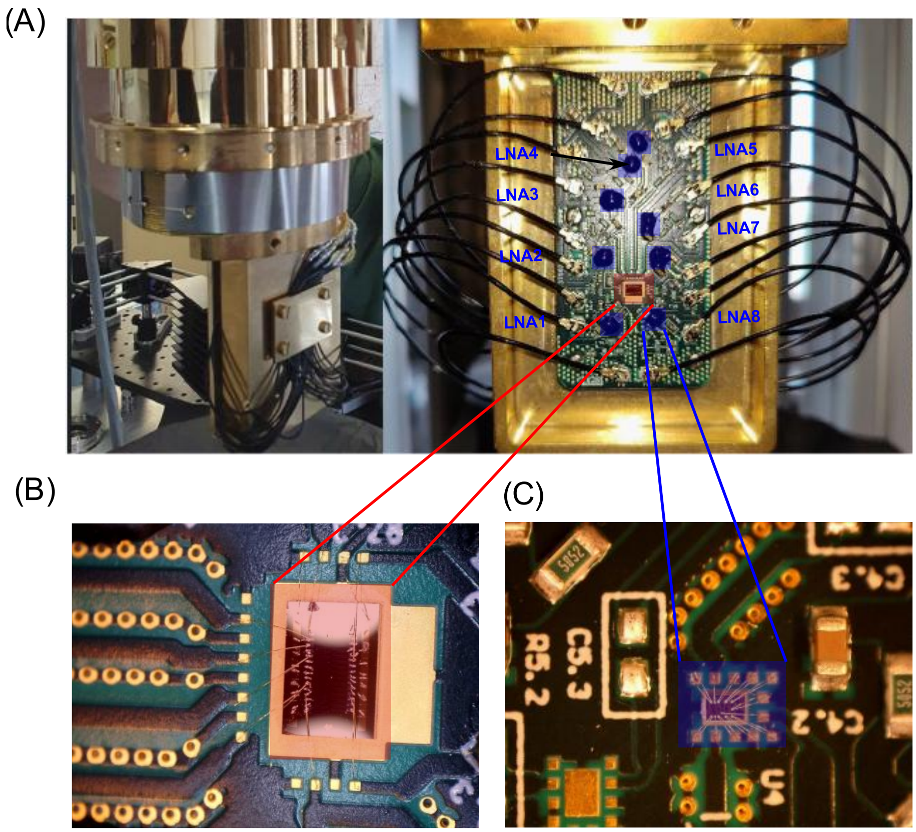

Figure S7: Cryogenic low-noise amplifiers (LNAs) integrated with the SSPD chip.

Each SNWD is individually biased by a current drive at room temperature. (**A)** shows the eight-pixel readout printed circuit board (PCB) mounted onto the 3.7 K stage, as well as the biasing lines that consist of two bias lines per pixel and two supply wires for the LNAs, totaling 18 leads**. (B)** Detector D_1 array, mounted onto the prototype PCB. **(C)** Wire bonded LNA unit.

7. Photocleavage QMS-SSPD spectra

Photochemistry in the gas phase has attracted recent interest as it promises to change the charge of proteins optically in free flight in high vacuum (45). It may even eventually allow the neutralization and reionization of proteins. In a proof-of-concept experiment, we here demonstrate the use of QMS-SSPD mass spectrometry to analyze the photochemistry of a tailored photocleavable Insulin compound that we had modified using a Ru-complex. The tagged compound and the cleavage mechanism are shown in **Figure S8**.

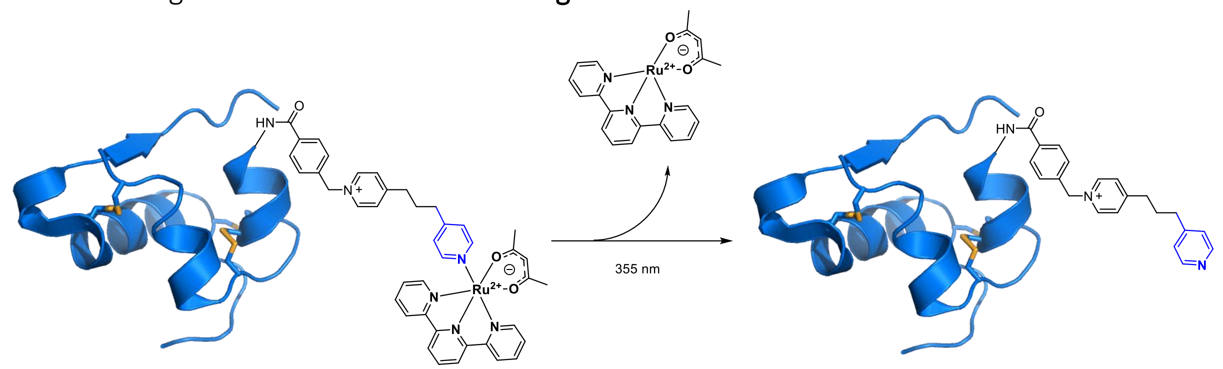

Figure S8: Mechanism for photo-cleavage of Ru-complex modified Insulin.

The functionalized insulin molecules were electrosprayed, guided into the quadrupole mass filter and detected by the SSPD.
Different to prior experiments (**Figure 2**), we have added a UV laser beam (355 nm, 500 kHz, 80 µJ pulse energy, 10 ps pulse duration) to induce photo-cleavage of the tailored Ru-tag (**Figure 1**). **Figure S9** shows the effect of that laser in the QMS-SSPD spectrum as the appearance of a new peak at Ins^{5+} (emerging from $[InsRu]^{6+}$) as well as a small peak at Ins^{6+}.

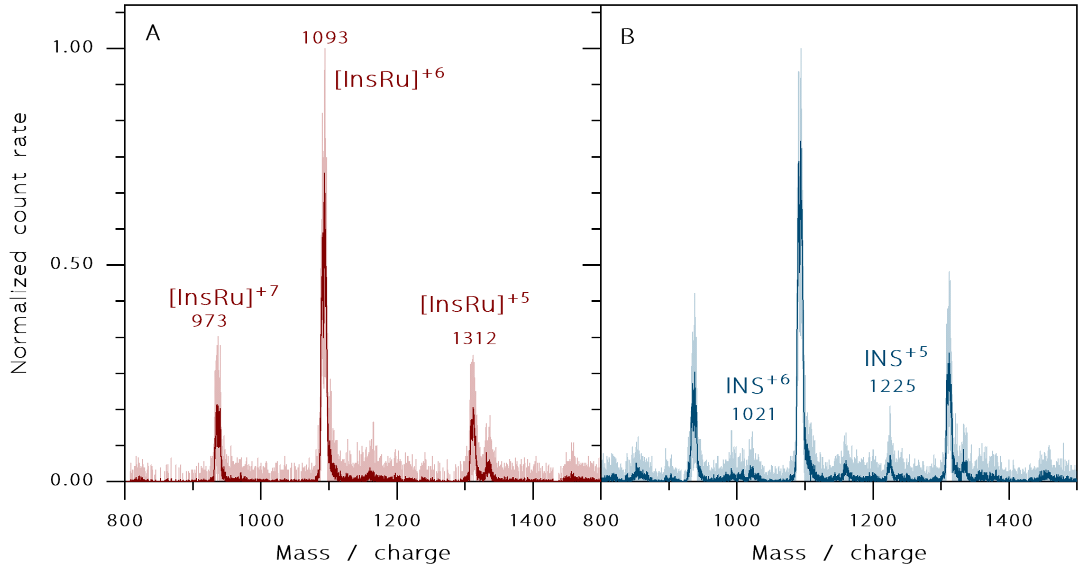

Figure S9: Photo-activated QMS-SSPD spectrum of Ru-complex modified Insulin.
An QMS-SSPD spectrum of Ru-complex modified Insulin, recorded (A) without and (B) with exposure to a laser beam at a pulse energy of 80 µJ at 355 nm is shown. The continuous nature of the detector fits the quasi-continuous laser interaction at 500 kHz. The appearance of the charge reduced protein Ins^{5+} and Ins^{6+}. The data was smoothed using a 10-point moving average.

8. Synthesis and verification of the molecular compound

Synthesis of Ru-complex modified insulin

Ru-complex (6.0 mg, 6.0 µmol), TSTU (2.2 mg, 7.2 µmol) and DIPEA (4.2 µl, 24 µmol) were dissolved in 0.5 ml DMF under N2 with stirring for 1 h at room temperature. Then the reaction mixture was added into insulin (35 mg, 6.0 µmol) and DIPEA (4.2 µl, 24 µmol) in 8 ml DMF, keep it stirring overnight at room temperature. After purification by preparative HPLC, a purple solid was obtained (4.2 mg).

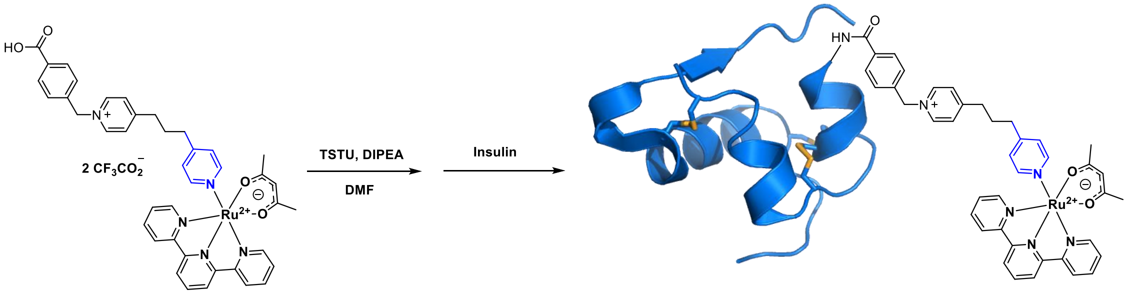

Figure S10: Synthesis of Ru-complex modified Insulin.

Verification of the Ru-complex modified insulin by mass spectrometry and NMR

Mass-to charge ratio m/z: calculated [M]: 6554.37, found [M]: 6554.53.

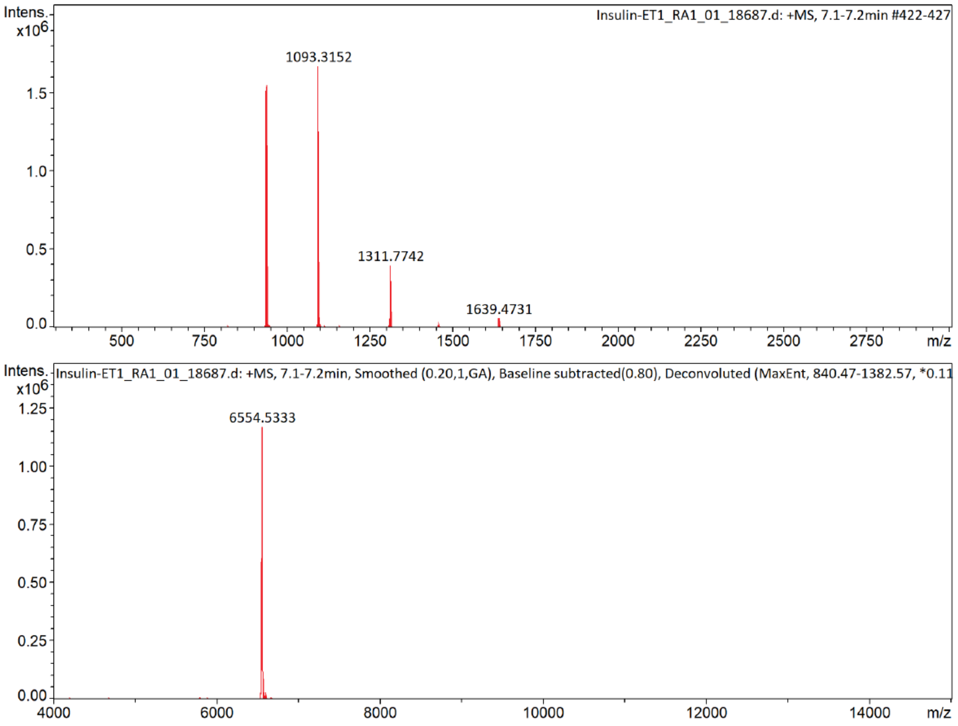

Figure S11: High resolution mass spectrum of the electrosprayed insulin compound.

Ru-complex: 1H NMR (500 MHz, CD3CN) δ 8.54 (d, J = 6.8 Hz, 2H), 8.52 – 8.49 (m, 2H), 8.44 – 8.30 (m, 4H), 8.04 (d, J = 8.4 Hz, 2H), 8.00 – 7.94 (m, 2H), 7.82 (t, J = 8.0 Hz, 1H), 7.78 (d, J = 6.7 Hz, 2H), 7.74 (d, J = 6.8 Hz, 2H), 7.59 (ddd, J = 7.6, 5.5, 1.3 Hz, 2H), 7.45 (d, J = 8.7 Hz, 2H), 6.83 (d, J = 6.8 Hz, 2H), 5.67 (s, 2H), 5.44 (s, 1H), 2.85 – 2.75 (m, 2H), 2.54 – 2.46 (m, 2H), 2.43 (s, 3H), 1.84 – 1.71 (m, 2H), 1.35 (s, 3H). 13C NMR (126 MHz, CD3CN) δ 188.63, 188.11, 167.10, 164.47, 161.85, 160.19, 152.54, 152.32, 151.58, 144.81, 138.71, 137.97, 132.52, 131.55, 131.39, 129.86, 129.21, 128.44, 125.62, 124.13, 123.05, 99.98, 63.96, 35.46, 34.21, 30.10, 28.62, 27.14. HRMS-ESI m/z: calcd. [M-H]+: 766.1973, found [M-H]+: 766.1976.

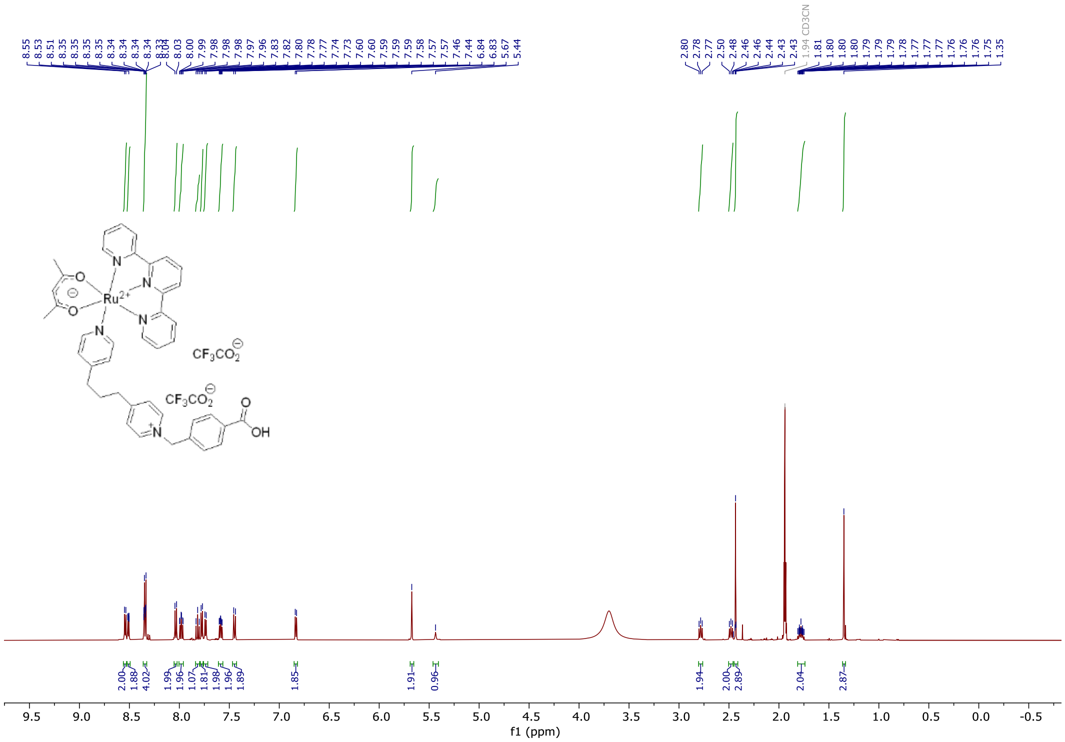

Figure S12: ^1H NMR (500 MHz, CD_3CN) of Ru-complex.

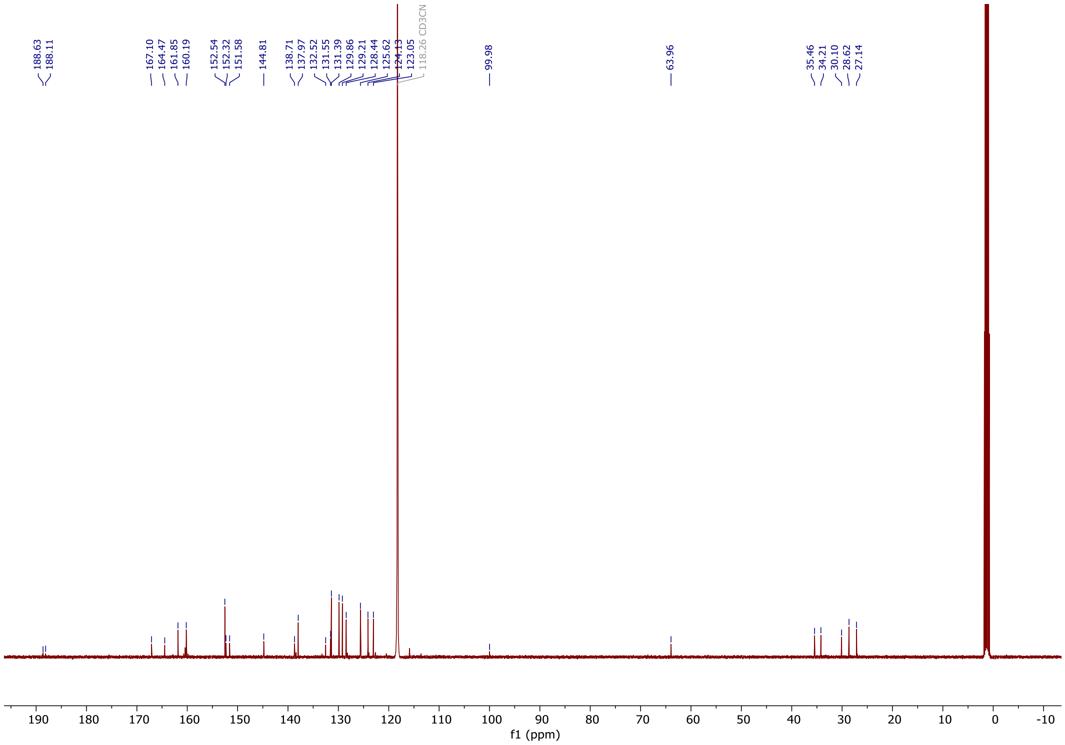

Figure S13: ^{13}C NMR (126 MHz, CD_3CN) of Ru-complex.

REFERENCES AND NOTES

1. W. Paul, Electromagnetic traps for charged and neutral particles. *Rev. Mod. Phys.* **62**, 531–540 (1990).

2. K. Fehre, D. Trojanowskaja, J. Gatzke, M. Kunitski, F. Trinter, S. Zeller, L. P. H. Schmidt, J. Stohner, R. Berger, A. Czasch, O. Jagutzki, T. Jahnke, R. Dorner, M. S. Schoffler, Absolute ion detection efficiencies of microchannel plates and funnel microchannel plates for multi-coincidence detection. *Rev. Sci. Instrum.* **89**, 045112 (2018).

3. A. Brunelle, P. Chaurand, S. Della-Negra, Y. L. Beyec, E. Parilis, Secondary electron emission yields from a csi surface under impacts of large molecules at low velocities ($5x10^3$–$7x10^4$ ms^{-1}). *Rap. Comm. Mass Spectr.* **11**, 353–362 (1997).

4. R. J. Beuhler, L. Friedman, Threshold studies of secondary electron emission induced by macro-ion impact on solid surfaces. *Nucl. Instr. Meth. Phys. Res. A* **170**, 309–315 (1980).

5. D. Twerenbold, D. Gerber, D. Gritti, Y. Gonin, A. Netuschill, F. Rossel, D. Schenker, J.-L. Vuilleumier, Single molecule detector for mass spectrometry with mass independent detection efficiency. *Proteomics* **1**, 66–69 (2001).

6. H. M. Berman, K. Henrick, H. Nakamura, Announcing the worldwide protein data bank. *Nat. Struct. Biol.* **10**, 980 (2003).

7. J. Lee, H. Chen, T. Liu, C. E. Berkman, P. T. Reilly, High resolution time-of-flight mass analysis of the entire range of intact singly-charged proteins. *Anal. Chem.* **83**, 9406–9412 (2011).

8. M. F. Jarrold, Peptides and proteins in the vapor phase. *Annu. Rev. Phys. Chem.* **51**, 179–207 (2000).

9. G. C. Hilton, J. Martinis, D. A.Wollman, K. D. Irwin, L. L. Dulcie, D. Gerber, P. M. Gillevet, D. Twerenbold, Impact energy measurement in time-of-flight mass spectrometry with cryogenic microcalorimeters. *Nature* **391**, 672–675 (1998).

10. K. D. Irwin, An application of electrothermal feedback for high resolution cryogenic particle detection. *Appl. Phys. Lett.* **66**, 1998–2000 (1995).

11. K. D. Irwin, G. C. Hilton, Transition-edge sensors, in *Cryogenic Particle Detection*, C. Enss, Ed. (Springer-Verlag, 2005), vol. 99, pp. 63–149.

12. A. J. Kerman, E. A. Dauler, W. E. Keicher, J. K. W. Yang, K. K. Berggren, G. Gol’tsman, B. Voronov, Kinetic-inductance-limited reset time of superconducting nanowire photon counters. *Appl. Phys. Lett.* **88**, 111116 (2006).

13. G. H. Wood, B. L. White, Pulses induced in tunneling currents between superconductors by alpha-particle bombardment. *Appl. Phys. Lett.* **15**, 237–239 (1969).

14. D. Twerenbold, J. L. Vuilleumier, D. Gerber, A. Tadsen, B. van den Brandt, P. M. Gillevet, Detection of single macromolecules using a cryogenic particle detector coupled to a biopolymer mass spectrometer. *Appl. Phys. Lett.* **68**, 3503–3505 (1996).

15. A. D. Semenov, G. N. Gol'tsman, A. A. Korneev, Quantum detection by current carrying superconducting film. *Phys. C. Supercond.* **351**, 349–356 (2001).

16. M. Ohkubo, Superconducting detectors for particles from atoms to proteins. *Phys. C. Supercond.* **468**, 1987–1991 (2008).

17. R. Cristiano, M. Ejrnaes, A. Casaburi, N. Zen, M. Ohkubo, Superconducting nano-strip particle detectors. *Supercond. Sci. Technol.* **28**, 124004 (2015).

18. W. H. Pernice, C. Schuck, O. Minaeva, M. Li, G. N. Goltsman, A. V. Sergienko, H. X. Tang, High-speed and high-efficiency travelling wave single-photon detectors embedded in nanophotonic circuits. *Nat. Commun.* **3**, 1325 (2012).

19. B. Korzh, Q.-Y. Zhao, J. P. Allmaras, S. Frasca, T. M. Autry, E. A. Bersin, A. D. Beyer, R. M. Briggs, B. Bumble, M. Colangelo, G. M. Crouch, A. E. Dane, T. Gerrits, A. E. Lita, F. Marsili, G. Moody, C. Peña, E. Ramirez, J. D. Rezac, N. Sinclair, M. J. Stevens, A. E. Velasco, V. B. Verma, E. E. Wollman, S. Xie, D. Zhu, P. D. Hale, M. Spiropulu, K. L. Silverman, R. P. Mirin, S. W. Nam, A. G. Kozorezov, M. D. Shaw, K. K. Berggren, Demonstration of sub-3 ps temporal resolution with a superconducting nanowire single-photon detector. *Nat. Photon.* **14**, 250–255 (2020).

20. I. E. Zadeh, J. Chang, J. W. N. Los, S. Gyger, S. Steinhauer, S. N. Dorenbos, V. Zwiller, Superconducting nanowire single-photon detectors: A perspective on evolution, state-of-the-art, future developments, and applications. *Appl. Phys. Lett.* **118**, 190502 (2021).

21. V. B. Verma, B. Korzh, A. B. Walter, A. E. Lita, R. M. Briggs, M. Colangelo, Y. Zhai, E. E. Wollman, A. D. Beyer, J. P. Allmaras, H. Vora, D. Zhu, E. Schmidt, A. G. Kozorezov, K. K. Berggren, R. P. Mirin, S. W. Nam, M. D. Shaw, Single-photon detection in the mid-infrared up to 10 μ m wavelength using tungsten silicide superconducting nanowire detectors. *Apl Photonics* **6**, 10.1063/5.0048049 (2021).

22. A. Divochiy, F. Marsili, D. Bitauld, A. Gaggero, R. Leoni, F. Mattioli, A. Korneev, V. Seleznev, N. Kaurova, O. Minaeva, G. Gol'tsman, K. G. Lagoudakis, M. Benkhaoul, F. Lévy, A. Fiore, Superconducting nanowire photon-number-resolving detector at telecommunication wavelengths. *Nat. Photon.* **2**, 302–306 (2008).

23. M. Giustina, M. A. M. Versteegh, S. Wengerowsky, J. Handsteiner, A. Hochrainer, K. Phelan, F. Steinlechner, J. Kofler, J.-Å. Larsson, C. Abellán, W. Amaya, V. Pruneri, M. W. Mitchell, J. Beyer, T. Gerrits, A. E. Lita, L. K. Shalm, S. W. Nam, T. Scheidl, R. Ursin, B. Wittmann, A. Zeilinger, Significant loophole-free test of Bell's theorem with entangled photons. *Phys. Rev. Lett.* **115**, 250401 (2015).

24. L. K. Shalm, E. Meyer-Scott, B. G. Christensen, P. Bierhorst, M. A. Wayne, M. J. Stevens, T. Gerrits, S. Glancy, D. R. Hamel, M. S. Allman, K. J. Coakley, S. D. Dyer, C. Hodge, A. E. Lita, V. B. Verma, C. Lambrocco, E. Tortorici, A. L. Migdall, Y. Zhang, D. R. Kumor, W. H. Farr, F. Marsili, M. D. Shaw, J. A. Stern, C. Abellán, W. Amaya, V. Pruneri, T. Jennewein, M. W. Mitchell, P. G. Kwiat, J. C. Bienfang, R. P. Mirin, E. Knill, S. W. Nam, Strong loophole-free test of local realism. *Phys. Rev. Lett.* **115**, 250402 (2015).

25. H. Takesue, S. W. Nam, Q. Zhang, R. H. Hadfield, T. Honjo, K. Tamaki, Y. Yamamoto, Quantum key distribution over a 40-dB channel loss using superconducting single-photon detectors. *Nat. Photon.* **1**, 343–348 (2007).

26. M. Aker, A. Beglarian, J. Behrens, A. Berlev, U. Besserer, B. Bieringer, F. Block, S. Bobien, M. Böttcher, B. Bornschein, L. Bornschein, T. Brunst, T. S. Caldwell, R. M. D. Carney, L. La Cascio, S.

Chilingaryan, W. Choi, K. Debowski, M. Deffert, M. Descher, D. Díaz Barrero, P. J. Doe, O. Dragoun, G. Drexlin, K. Eitel, E. Ellinger, R. Engel, S. Enomoto, A. Felden, J. A. Formaggio, F. M. Fränkle, G. B. Franklin, F. Friedel, A. Fulst, K. Gauda, W. Gil, F. Glück, R. Grössle, R. Gumbsheimer, V. Gupta, T. Höhn, V. Hannen, N. Haußmann, K. Helbing, S. Hickford, R. Hiller, D. Hillesheimer, D. Hinz, T. Houdy, A. Huber, A. Jansen, C. Karl, F. Kellerer, J. Kellerer, M. Kleifges, M. Klein, C. Köhler, L. Köllenberger, A. Kopmann, M. Korzeczek, A. Kovalík, B. Krasch, H. Krause, N. Kunka, T. Lasserre, T. L. Le, O. Lebeda, B. Lehnert, A. Lokhov, M. Machatschek, E. Malcherek, M. Mark, A. Marsteller, E. L. Martin, C. Melzer, A. Menshikov, S. Mertens, J. Mostafa, K. Müller, H. Neumann, S. Niemes, P. Oelpmann, D. S. Parno, A. W. P. Poon, J. M. L. Poyato, F. Priester, S. Ramachandran, R. G. H. Robertson, W. Rodejohann, M. Röllig, C. Röttele, C. Rodenbeck, M. Ryšavý, R. Sack, A. Saenz, P. Schäfer, A. Schaller nee Pollithy, L. Schimpf, K. Schlösser, M. Schlösser, L. Schlüter, S. Schneidewind, M. Schrank, B. Schulz, A. Schwemmer, M. Šefčík, V. Sibille, D. Siegmann, M. Slezák, F. Spanier, M. Steidl, M. Sturm, M. Sun, D. Tcherniakhovski, H. H. Telle, L. A. Thorne, T. Thümmler, N. Titov, I. Tkachev, K. Urban, K. Valerius, D. Vénos, A. P. V. Hernández, C. Weinheimer, S. Welte, J. Wendel, J. F. Wilkerson, J. Wolf, S. Wüstling, J. Wydra, W. Xu, Y. R. Yen, S. Zadoroghny, G. Zeller, Direct neutrino-mass measurement with sub-electronvolt sensitivity. *Nat. Phys.* **18**, 160–166 (2022).

27. M. Mirhosseini, A. Sipahigil, M. Kalaee, O. Painter, Superconducting qubit to optical photon transduction. *Nature* **588**, 599–603 (2020).

28. E. E. Wollman, V. B. Verma, A. E. Lita, W. H. Farr, M. D. Shaw, R. P. Mirin, S. W. Nam, Kilopixel array of superconducting nanowire single-photon detectors. *Opt. Express* **27**, 35279–35289 (2019).

29. L. Chen, D. Schwarzer, V. B. Verma, M. J. Stevens, F. Marsili, R. P. Mirin, S. W. Nam, A. M. Wodtke, Mid-infrared laser-induced fluorescence with nanosecond time resolution using a superconducting nanowire single-photon detector: New technology for molecular science. *Acc. Chem. Res.* **50**, 1400–1409 (2017).

30. K. Suzuki, S. Miki, S. Shiki, Z. Wang, M. Ohkubo, Time resolution improvement of superconducting nbn stripline detectors for time-of-flight mass spectrometry. *Appl. Phys. Express* **1**, 031702 (2008).

31. K. Suzuki, S. Miki, Z. Wang, Y. Kobayashi, S. Shiki, M. Ohkubo, Superconducting NbN thin-film nanowire detectors for time-of-flight mass spectrometry. *J. Low. T. Phys.* **151**, 766–770 (2008).

32. K. Suzuki, S. Shiki, M. Ukibe, M. Koike, S. Miki, Z. Wang, M. Ohkubo, Hot-spot detection model in superconducting nano-stripline detector for keV ions. *Appl. Phys. Express* **4**, 083101 (2011).

33. M. Marksteiner, A. Divochiy, M. Sclafani, P. Haslinger, H. Ulbricht, A. Korneev, A. Semenov, G. Gol'tsman, M. Arndt, A superconducting NbN detector for neutral nanoparticles. *Nanotechnology* **20**, 455501 (2009).

34. J. B. Fenn, M. Mann, C. K. Meng, S. F. Wong, C. M. Whitehouse, Electrospray ionization for mass spectrometry of large biomolecules. *Science* **246**, 64–71 (1989).

35. D. D. Ebeling, M. S. Westphall, M. Scalf, L. M. Smith, Corona discharge in charge reduction electrospray mass spectrometry. *Anal. Chem.* **72**, 5158–5161 (2000).

36. R. Gourgues, J. W. N. Los, J. Zichi, J. Chang, N. Kalhor, G. Bulgarini, S. N. Borenbos, V. Zwiller, I. E. Zadeh, Superconducting nanowire single photon detectors operating at temperature from 4 to 7 K. *Opt. Express* **27**, 24601–24609 (2019).

37. A. Verevkin, J. Zhang, R. Sobolewski, A. Lipatov, O. Okunev, G. Chulkova, A. Korneev, K. Smirnov, G. Gol’tsman, A. Semenov, Detection efficiency of large-active-area nbn single-photon superconducting detectors in the ultraviolet to near-infrared range. *Appl. Phys. Lett.* **80**, 4687–4689 (2002).

38. A. I. Bezuglyj, V. A. Shklovskij, B. Budinská, B. Aichner, V. M. Bevz, M. Y. Mikhailov, D. Y. Vodolazov, W. Lang, O. V. Dobrovolskiy, Vortex jets generated by edge defects in current-carrying superconductor thin strips. *Phys. Rev. B* **105**, 214507 (2022).

39. M. Sidorova, A. D. Semenov, H. W. Hübers, S. Gyger, S. Steinhauer, X. Zhang, A. Schilling, Magnetoconductance and photoresponse properties of disordered nbtin films. *Phys. Rev. B* **104**, 184514 (2021).

40. D. Y. Vodolazov, Single-photon detection by a dirty current-carrying superconducting strip based on the kinetic-equation approach. *Phys. Rev. Appl.* **7**, 034014 (2017).

41. M. Sclafani, M. Marksteiner, F. M. Keir, A. Divochiy, A. Korneev, A. Semenov, G. Gol'tsman, M. Arndt, Sensitivity of a superconducting nanowire detector for single ions at low energy. *Nanotechnology* **23**, 065501 (2012).

42. R. Liu, Q. Li, L. M. Smith, Detection of large ions in time-of-flight mass spectrometry: Effects of ion mass and acceleration voltage on microchannel plate detector response. *J. Am. Soc. Mass Spectrom.* **25**, 1374–1383 (2014).

43. K. Anggara, H. Ochner, S. Szilagyi, L. Malavolti, S. Rauschenbach, K. Kern, Landing proteins on graphene trampoline preserves their gas-phase folding on the surface. *ACS Cent. Sci.* **9**, 151–158 (2023).

44. S. Rauschenbach, R. Vogelgesang, N. Malinowski, J. W. Gerlach, M. Benyoucef, G. Costantini, Z. Deng, N. Thontasen, K. Kern, Electrospray ion beam deposition: Soft-landing and fragmentation of functional molecules at solid surfaces. *ACS Nano* **3**, 2901–2910 (2009).

45. Y. Hua, M. Strauss, S. Fisher, M. F. X. Mauser, P. Manchet, M. Smacchia, P. Geyer, A. Shayeghi, M. Pfeffer, T. H. Eggenweiler, S. Daly, J. Commandeur, M. Mayor, M. Arndt, T. Šolomek, V. Köhler, Giving the green light to photochemical uncaging of large biomolecules in high vacuum. *JACS Au*. **3**, 2790–2799 (2023).

46. M. Ejrnaes, A. Casaburi, O. Quaranta, S. Marchetti, A. Gaggero, F. Mattioli, R. Leoni, S. Pagano, R. Cristiano, Characterization of parallel superconducting nanowire single photon detectors. *Supercond. Sci. Technol.* **22**, 055006 (2009).

47. R. Cristiano, A. Casaburi, E. Esposito, M. Ejrnaes, S. Pagano, K. Suzuki, N. Zen, M. Ohkubo, Parallel superconducting strip-line detectors for time-of-flight mass spectrometry. *J. Low Temp. Phys.* **167**, 979–984 (2012).
